# Regulatory Mechanism of Exogenous ABA on Gibberellin Signaling and Antioxidant Responses in *Rhododendron chrysanthum* Pall. Under UV-B Stress

**DOI:** 10.3390/ijms252413651

**Published:** 2024-12-20

**Authors:** Wang Yu, Kun Cao, Hongwei Xu, Xiaofu Zhou

**Affiliations:** Jilin Provincial Key Laboratory of Plant Resource Science and Green Production, Jilin Normal University, Siping 136000, China; 15043512434@163.com (W.Y.); kun2199@163.com (K.C.)

**Keywords:** metabolomics, transcriptomics, *R. chrysanthum*, UV-B, plant hormone

## Abstract

In the present work, we examined the effects of exogenous abscisic acid (ABA) under ultraviolet B (UV-B) exposure on gibberellin (GA) production, signaling, and antioxidant-related genes in *Rhododendron chrysanthum* Pall (*R. chrysanthum*). Using transcriptomics, acetylated proteomics, and widely targeted metabolomics, the effects of UV-B stress on *R. chrysanthum* and the regulatory effects of exogenous ABA on it were revealed from multiple perspectives. The findings revealed that *R. chrysanthum*’s antioxidant enzyme genes were differentially expressed by UV-B radiation and were substantially enriched in the glutathione metabolic pathway. Exogenous ABA supplementation boosted plant resistance to UV-B damage and further enhanced the expression of antioxidant enzyme genes. Furthermore, under UV-B stress, glutathione reductase, glutathione peroxidase, and L-ascorbate peroxidase were found to be the primary antioxidant enzymes controlled by exogenous ABA. In addition, gibberellin content was altered due to UV-B and exogenous ABA treatments, with greater effects on GA3 and GA53. The acetylation proteomics study’s outcomes disclosed that the three main oxidative enzymes’ acetylation modifications were dramatically changed during UV-B exposure, which may have an impact on the antioxidant enzymes’ functions and activities. The protective impact of exogenous ABA and gibberellin on *R. chrysanthum’*s photosynthetic system was further established by measuring the parameters of chlorophyll fluorescence. This research offers a theoretical foundation for the development of breeding highly resistant plant varieties as well as fresh insights into how hormone levels and antioxidant systems are regulated by plants in response to UV-B damage.

## 1. Introduction

Ultraviolet radiation from sunlight has significant effects on organisms in the Earth’s ecosystems [1]. Based on their wavelength range, UV radiation is divided into three primary bands: shortwave UV (UV-C, 200–280 nm), midwave UV (UV-B, 280–315 nm), and longwave UV (UV-A, 315–400 nm) [2,3]. Among the three types of ultraviolet radiation, UV-B is the band found in nature that has the greatest impact on living organisms [3,4,5]. Plants are negatively impacted by increased UV-B radiation resulting from ozone layer depletion. This damage includes apoptosis, pigment degradation, obstruction of photosynthesis and metabolic pathways, and damage to biomolecules [6,7,8]. In this sense, different ground-based plants have progressively evolved their own distinctive adaptation mechanisms in response to UV-B damage in order to live.

*Rhododendron chrysanthum* Pall (*R. chrysanthum*), belonging to the genus Rhododendron in the family Rhododendronaceae, is an evergreen shrub or small tree [9]. This plant can endure the severe conditions found in Changbai Mountain, including low temperatures, UV radiation, and other environmental stresses. *R. chrysanthum’*s ability to withstand UV-B radiation was demonstrated by research involving UV-B resistance, which allowed the plant to preserve its regular existence by causing notable alterations in its metabolism linked to amino acids [10]. Research on acetylation proteomics revealed that *R. chrysanthum* reduces UV-B-induced damage by acetylating proteins involved in photosystem II and glycolytic processes [11,12]. In actuality, plants’ natural antioxidant system can also react to UV-B stress. Numerous research has shown the distinct coping mechanisms of *R. chrysanthum*. Although transcriptomics and metabolomics play important roles in the study of plant response to environmental stresses, there is still a relative paucity of studies targeting the antioxidant gene response of *R. chrysanthum* under UV-B stress. Although studies have been conducted to explore the acetylation of proteins associated with *R. chrysanthum*, studies on the acetylation modification of antioxidant enzymes regulated by antioxidant genes are currently very limited. Therefore, an in-depth study of their acetylation modifications is important for understanding the stress mechanisms of *R. chrysanthum*.

In plant cells, UV-B stress can induce the production of excessive reactive oxygen species (ROS), such as superoxide anion and hydrogen peroxide (H_2_O_2_) [13,14]. These excess ROS damage biomolecules such as DNA, proteins and lipids in plant cells, leading to cellular dysfunction [15]. Plants have a complex antioxidant system that guards against damage from ROS. This system is made up of both non-enzymatic antioxidants like ascorbic acid, glutathione, and carotenoids, as well as a range of antioxidant enzymes like superoxide dismutase (SOD), catalase (CAT), and glutathione peroxidase (GPX) [16,17]. Upon UV-B stress, these antioxidant genes respond rapidly to modulate the antioxidant system and produce antioxidants [18]. These antioxidants shield plant cells from oxidative harm by scavenging excess reactive oxygen species.

A crucial phytohormone in plants, abscisic acid (ABA) is involved in both the growth and development of plants as well as their reaction to stressors such as adversity. Applying exogenous ABA to plants increases their resilience to a range of environmental challenges [19]. According to some research, ABA spraying can boost the carbon metabolism of sweet potato leaves, increase yield, and improve photosynthetic capability [20]. Key enzymes involved in the production of starch were found to be more active in rice grains treated with exogenous ABA, which also encouraged the buildup of starch [21]. Furthermore, by controlling the expression of genes or enzymes involved in hormone production, metabolism, and signaling, exogenous ABA can modify the levels of endogenous hormones [22]. Gibberellin is likewise an important class of phytohormones and belongs to the diterpenoid class [23]. Enhancing a plant’s resistance to adverse stressors is another function of gibberellins [24]. In some studies, it has been found that gibberellins are able to influence photosynthesis in plants, helping them to better utilize light energy under adverse conditions by regulating photosynthetic efficiency [25]. Gibberellins interact with other hormones in plants, like ethylene and ABA, to control how the plant reacts to adversity [26,27]. Importantly, the DELLA protein, a key factor in the GA signaling pathway, is coregulated by various hormones such as GA itself, jasmonate, ethylene, and ABA, thus exercising a corresponding regulatory role [20]. Studies on the exogenous ABA control of *R. chrysanthum* genes associated with antioxidants are scarce, nevertheless. Similar to this, not much research has been conducted on how exogenous ABA regulates GA-related pathways of *R. chrysanthum* exposed to UV-B.

In summary, in order to fill the research gap in the study of how exogenous ABA regulates antioxidant enzymes and metabolic pathways in *R. chrysanthum* in response to UV-B stress, the present experiment was conducted to analyze the mechanism of regulation of gibberellin biosynthesis as well as signaling-related genes of *R. chrysanthum* under UV-B stress by exogenous ABA through transcriptomics as well as the effects of exogenous ABA on antioxidant-related genes under UV-B stress. Combined with the results of targeted metabolomics, the important role played by the enzymes regulated by these key genes in the antioxidant metabolic pathway was confirmed. Post-translational modifications of key antioxidant enzymes in *R. chrysanthum* were also explored using acetylated proteomics to show the effect of UV-B radiation on antioxidant enzymes. The results are important for understanding how exogenous ABA responds to environmental stress by regulating the antioxidant system of plants and provide a theoretical basis and molecular markers for the breeding of highly resilient plant varieties.

## 2. Results

### 2.1. UV-B and Exogenous ABA Induced Changes in the Content of GAs

**Figure 1:**
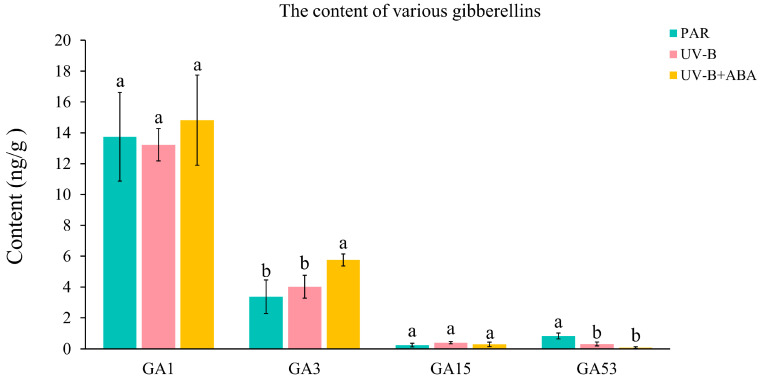
Changes in GA content under UV-B and exogenous ABA treatments. The data were analyzed by ANOVA. If the differences were significant, Duncan’s test was used to distinguish mean differences at the *p* < 0.05 level. Data are presented as means ± SD (standard deviation), based on three biological replicates (*n* = 3). Different lowercase letters denote statistically significant differences at *p* < 0.05 among the various treatments within the same group.

To investigate the effect of exogenous ABA on gibberellin (GA) content in *R. chrysanthum* under UV-B stress, GA content was examined using widely targeted metabolomics on PAR-treated, UV-B-treated, and UV-B-co-treated with exogenous ABA experimental materials. According to the data, UV-B and exogenous ABA had less of an effect on GA1, with UV-B decreasing its content and exogenous ABA increasing it. However, none of these changes differed significantly. Exogenous ABA caused a considerable rise in GA3 content after it was applied. Although UV-B also increased GA3 content, the difference was not significant compared to the PAR treatment. The changes in GA15 were less pronounced; UV-B caused little rise in content, whereas exogenous ABA treatment caused slight decrease. With UV-B radiation, the amount of GA53 was dramatically reduced; this reduction continued with exogenous ABA therapy (Figure 1).

This implies that exogenous ABA may have an impact on *R. chrysanthum*’s GAs content under UV-B. Furthermore, exogenous ABA therapy causes considerable alteration in the contents of GA3 and GA53, which is probably the main influencing factor. Thus, ABA and GAs of *R. chrysanthum* are able to interact and function together in response to UV-B stress.

### 2.2. Exogenous ABA and GA3 Improve R. chrysanthum’s Capacity for Photosynthetic Processes Under UV-B Stress

The above results showed that exogenous ABA was able to significantly elevate the content of GA3, suggesting that high content of GA3 may contribute to the enhancement of stress tolerance in *R. chrysanthum*. Therefore, in this experiment, chlorophyll fluorescence parameters were simultaneously measured after exogenous ABA and GA3 treatments to compare the roles played by ABA and gibberellin in resisting UV-B stress.

**Figure 2:**
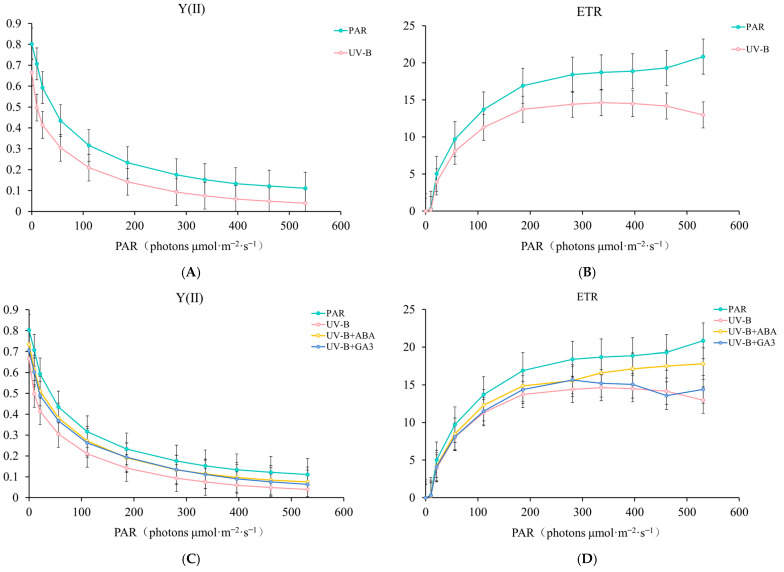

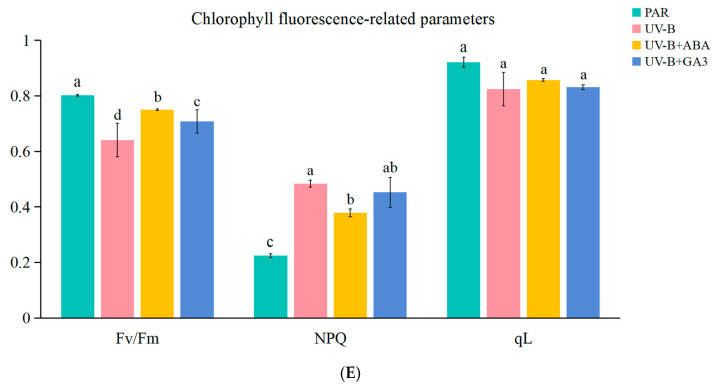
Changes in chlorophyll fluorescence parameters of UV-B with exogenous hormone treatments (ABA and GA3). (**A**) Y(II) fold plot of UV-B radiation treatment; (**B**) ETR fold plot of UV-B radiation treatment; (**C**) Y(II) fold plot of UV-B and exogenous hormone treatment; (**D**) ETR fold plot of UV-B and exogenous hormone treatment; (**E**) bar graph of photosynthetic performance indices for UV-B and exogenous hormone treatments. The data were analyzed by ANOVA. If the differences were significant, Duncan’s test was used to distinguish mean differences at the *p* < 0.05 level. Data are presented as means ± SD (standard deviation), based on three biological replicates (*n* = 3). Different lowercase letters denote statistically significant differences at *p* < 0.05 among the various treatments within the same group.

The photosynthetic ability of *R. chrysanthum* was lowered by UV-B radiation, as evidenced by the Y(II) (photochemical yield of PSII) and ETR (actual electron transport rate) curves following UV-B radiation being lower than those following PAR treatment (Figure 2A,B). On this basis, the Y(II) and ETR curves of the experimental materials treated with two exogenous hormones (ABA and GA3) were intermediate between PAR and UV-B treatments. This indicates that ABA and GA3 have a protective effect on the photosynthetic system of *R. chrysanthum* (Figure 2C,D).

The bar graph data showed that Fv/Fm (Maximal photochemical efficiency of PSII) decreased significantly after UV-B radiation compared to the PAR-treated group. The exogenous hormones (ABA and GA3) alleviated this trend, and Fv/Fm increased significantly. qL (photochemical quenching) and Fv/Fm showed similar trends, but these differences were not significant. The significant increase in NPQ (non-photochemical quenching) after UV-B radiation suggests that the photosynthetic system of *R. chrysanthum* was subjected to excess light energy and dissipated the excess in the form of heat energy. Similarly, this trend was alleviated after exogenous ABA and NPQ decreased significantly. This indicated that exogenous ABA enhanced the utilization of light energy by *R. chrysanthum*. A similar pattern of change was observed after exogenous GA3, but the effect was not as pronounced as that of exogenous ABA (Figure 2E).

Combined with the findings of exogenous ABA affects *R. chrysanthum’*s gibberellin content under UV-B radiation, it is evident that exogenous ABA controls the plant’s gibberellin-related pathway and collaborates with gibberellin to help the plant respond favorably to UV-B stress.

### 2.3. Differential Expression of R. chrysanthum Antioxidant Genes by Exogenous ABA Under UV-B Stress

The antioxidant system in plants is crucial for protecting against UV-B stress. Therefore, this experiment focused on exploring the changes in antioxidant enzyme genes between groups M (PAR treatment), N (UV-B treatment) and Q (UV-B co-treatment with exogenous ABA). The objective was to find important antioxidant enzymes and the genes that correspond to them in *R. chrysanthum* reacting to UV-B, as well as investigate how exogenous ABA regulates antioxidant genes under UV-B stress.

**Figure 3:**
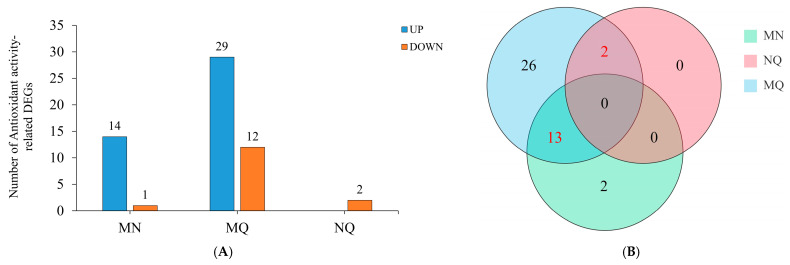

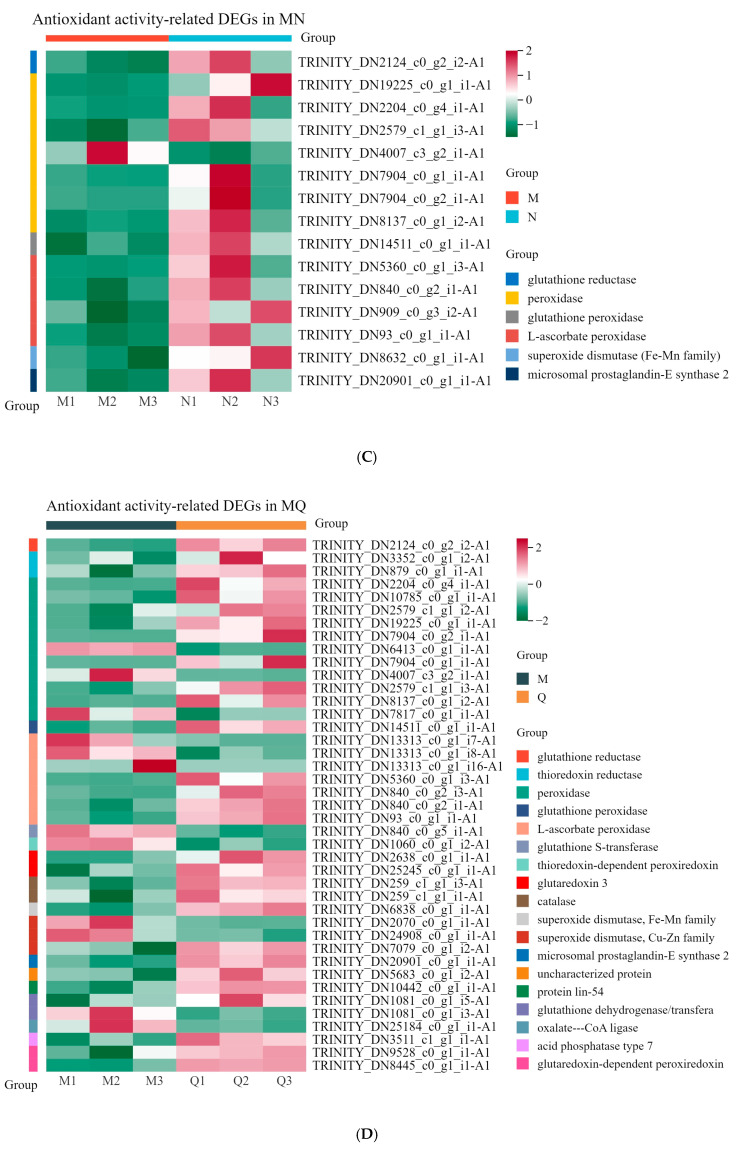

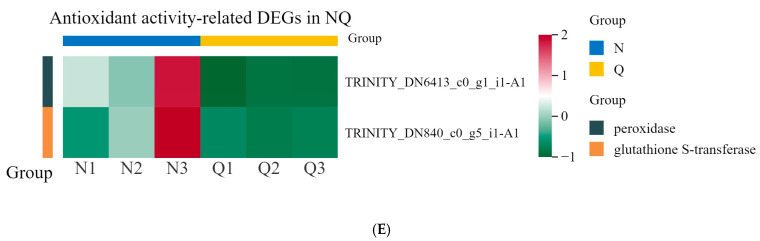
Alterations in DEGs linked to antioxidant activity in reacting to exogenous ABA and UV-B. (**A**) Statistics of the number of antioxidant enzyme-related DEGs in each comparative group (MN, NQ and MQ); (**B**) Wayne’s plots of antioxidant enzyme-related DEGs in each comparative group; (**C**–**E**) clustering heatmaps of antioxidant enzyme-related DEGs in each comparative group. The expression of relevant DEGs in the graphs is indicated by color. Redder color indicates higher relative expression, and greener color indicates lower.

The results of GO analysis showed that there were 15 antioxidant activity-related differentially expressed genes (DEGs) (14 upregulated and 1 downregulated) in MN (MvsN), 41 antioxidant activity-related DEGs (29 upregulated and 12 downregulated) in MQ (MvsQ), and 2 antioxidant activity-associated DEGs in NQ (NvsQ) (2 downregulated) (Figure 3A, Supplementary Tables S1–S3). Following UV-B radiation, the majority of genes associated with antioxidant enzymes were found to be upregulated, with more DEGs for peroxidase and L-ascorbate peroxidase, according to the clustered heat map data (Figure 3C). This implies that in reaction to UV-B exposure, *R. chrysanthum* activates its own antioxidant mechanism.

Similarly, under UV-B stress, exogenous ABA contributed to the upregulation of most of the DEGs associated with antioxidant enzymes in *R. chrysanthum* (Figure 3D). This shows that exogenous ABA may be able to further regulate the antioxidant system in order to shield *R. chrysanthum’*s photosynthetic system from harm. This phenomenon is also consistent with the results of the photosynthetic performance index. Of note was the downregulation of both DEGs in the NQ group (Figure 3E). The reason for this is that relative to PAR treatment, although both UV-B treatment and exogenous ABA resulted in the upregulation of antioxidant enzyme-related genes, the effect of UV-B radiation was greater.

Wayne plots were used to show the key antioxidant enzymes and their associated DEGs reacting to UV-B by displaying the pooled partial DEGs between the various comparison groups. The results showed that a total of 15 DEGs appeared in the pooled section between the various comparison groups (Figure 3B). The clustered heatmap shows the changes in expression of 15 DEGs associated with six key antioxidant enzymes. The genes related to key antioxidant enzymes such as glutathione peroxidase and glutathione reductase were upregulated after UV-B radiation, and these genes were further upregulated by exogenous ABA (Supplementary Figure S1). These 15 DEGs were considerably enriched in antioxidant-related pathways, notably glutathione metabolism and phenylpropanoid biosynthesis, as shown by KEGG enrichment data (Supplementary Figure S2).

### 2.4. Regulation of GA-Related Pathways by Exogenous ABA Under UV-B Stress

The variations of several DEGs in GA biosynthesis and signaling pathway were demonstrated by findings, which linked UV-B radiation and exogenous ABA treatment (Supplementary Figures S3 and S4). These DEGs ultimately led to significant changes in the GA content of *R. chrysanthum* and activated the GA signaling pathway.

**Figure 4:**
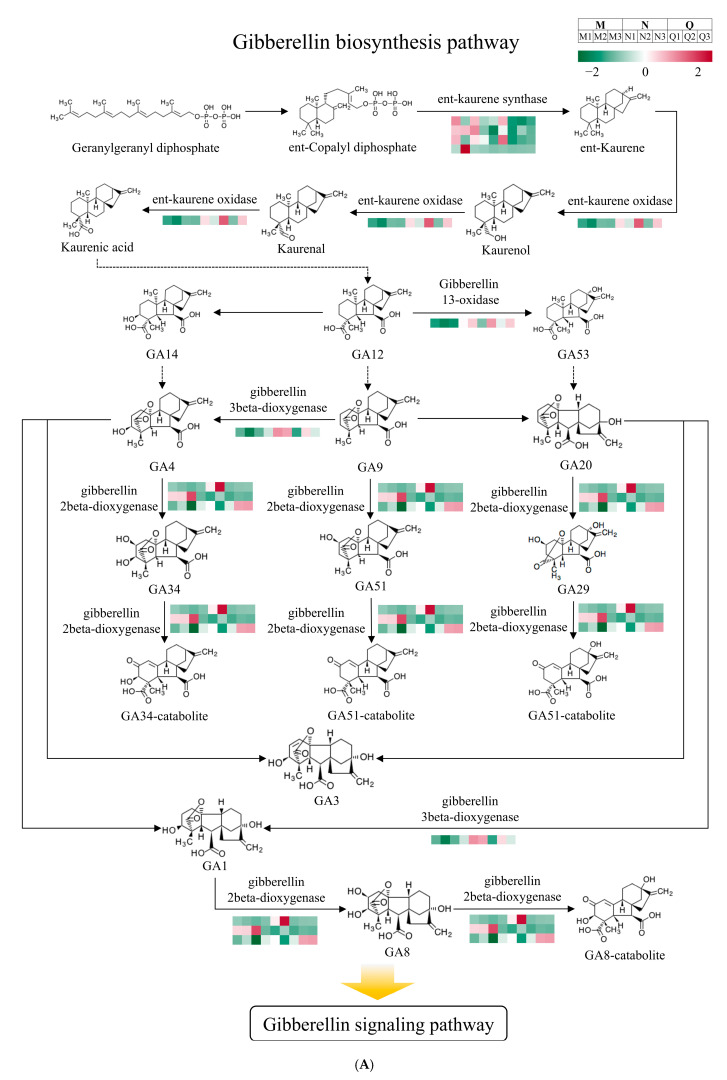

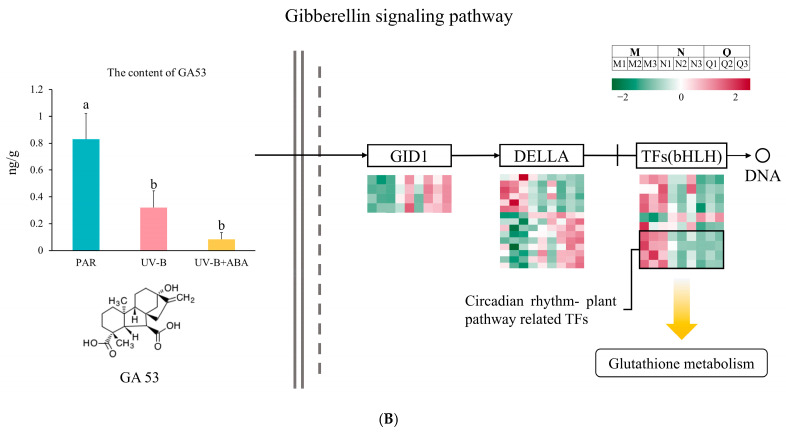
Diagram of the pattern of gibberellin biosynthesis and signaling pathways. (**A**) Diagram of the pattern of gibberellin biosynthesis pathway; (**B**) Diagram of the pattern of gibberellin signaling pathway. The expression of relevant DEGs in the graph is indicated by color. Redder colors indicate higher relative expression, and greener colors indicate lower expression. Solid arrows represent direct connections, and dashed arrows represent indirect connections. The standard deviation is indicated by error bars, whereas the mean of three biological replicates (*n* = 3) is represented by bar heights. Significant differences (*p* < 0.05) are indicated by different lowercase letters.

In the GA biosynthesis pathway, the genes for the key enzymes ent-kaurene synthase, ent-kaurene oxidase, and GA 2beta-dioxygenase were all differentially expressed (Figure 4A). Notably, DEGs in the pathways involved in GA3 biosynthesis were expressed higher in both groups N and Q than in group M (except for ent-kaurene synthase) (Figure 4A). Combined with the results of changes in GA content, exogenous ABA contributed to the upregulation of most DEGs in the GA3 biosynthesis pathway, which ultimately led to a significant increase in GA3 content.

For GA53, the DEGs in its biosynthetic pathway mostly overlap with GA3. Exogenous ABA eventually led to a significant upregulation of DEGs of GA 13-oxidase. Exogenous ABA not only significantly reduced the content of GA53, but also inhibited the activity of GA 13-oxidase by regulating its DEG. Therefore, exogenous ABA mainly regulated the biosynthesis of *R. chrysanthum* GA53 to turn on the downstream signaling pathway.

In the GA signaling pathway, GID1-involved DEGs are consistently upregulated under the influence of UV-B and ABA. The majority of DEGs involved in DELLA were further upregulated, which ultimately led to the negative regulatory role of the bHLH transcription factor in controlling downstream metabolic pathways (Figure 4B). Interestingly, some of these GA signaling pathway-regulated bHLH transcription factors appeared in the Circadian rhythm–plant pathway. This suggests that exogenous ABA is likely to regulate both the GA signaling pathway and the Circadian rhythm–plant pathway under UV-B stress, and that these two signaling pathways interact with each other to perform certain biological functions together.

### 2.5. Quality Control (QC) of Metabolomics and Classification of Detected Metabolites

**Figure 5:**
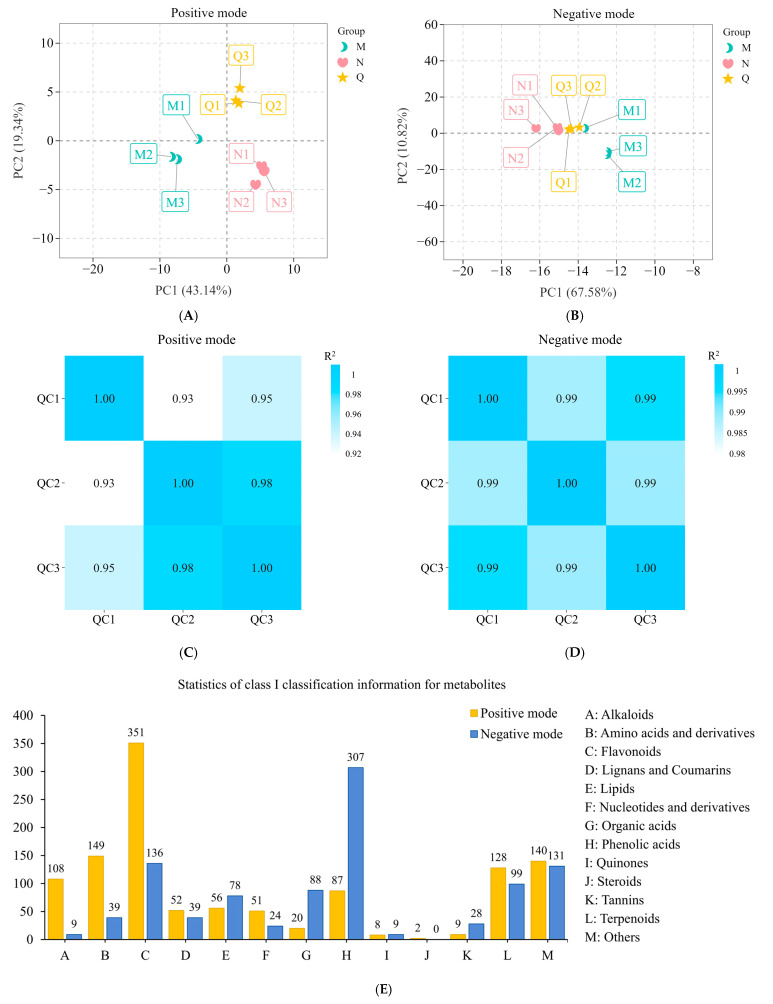
Quality assessment and annotation of broadly targeted metabolomics data. (**A**,**B**) PCA analysis of metabolites detected in different samples. PC1 and PC2 represent the first and second principal components, respectively. Different colored dots represent samples from different treatment groups; (**C**,**D**) stability assessment of the UPLC-MS/MS system. Quality control (QC) samples were prepared from a mixture of sample extracts and used to analyze the reproducibility of the samples under the same treatments. The Pearson’s correlation coefficient R2 between the QC samples was calculated using the relative quantitative values of the metabolites. The correlation between the QC samples was very strong (R2 close to 1), which indicates that the assay process is very stable, and the data quality is high. (**E**) Overview of class I information for metabolites. The height of the bars represents the number of metabolites. Positive and negative patterns represent different scanning methods.

In the PCA (principal component analysis) results of this study, samples within the same treatment group clustered together, indicating high reproducibility within each treatment group (Figure 5A,B). The high correlation between the quality control samples (QC) indicated that UPLC-MS/MS (ultra-performance liquid chromatography–tandem mass spectrometry) was highly stable (Figure 5C,D). OPLS-DA (orthogonal partial least squares–discriminant analysis) is designed to differentiate between different groups of samples and to identify key variables that affect group classification, i.e., differential metabolites. And the VIP threshold was set to 1 to screen for differential metabolites (DMs) (Supplementary Figure S5A,B). The Q^2^ of both detection modalities was greater than 0.5, as indicated by the model validation plot results, demonstrating the validity of the OPLS-DA data (Supplementary Figure S5C,D). The metabolomics data are trustworthy and appropriate for further metabolic pathway study, according to the aforementioned results.

A total of 2149 metabolites were detected by broadly targeted metabolomics in this study (Figure 5E). Among the known metabolites detected in the positive mode, flavonoids, amino acids and derivatives, and terpenoids predominated. Among the known metabolites detected in the negative mode, flavonoids, phenolic acids, and terpenoids predominated.

### 2.6. Modulation of Key Antioxidant Enzymes by Exogenous ABA in the Glutathione Metabolism Pathway

The above transcriptomics KEGG enrichment analysis results indicated that UV-B radiation and exogenous ABA mainly led to significant enrichment of antioxidant-related DEGs in the glutathione metabolism pathway (Supplementary Figure S2). Therefore, the present study synthesized the results of transcriptomics and metabolomics, focusing on the role played by key oxidative enzymes in the glutathione metabolism pathway.

**Figure 6:**
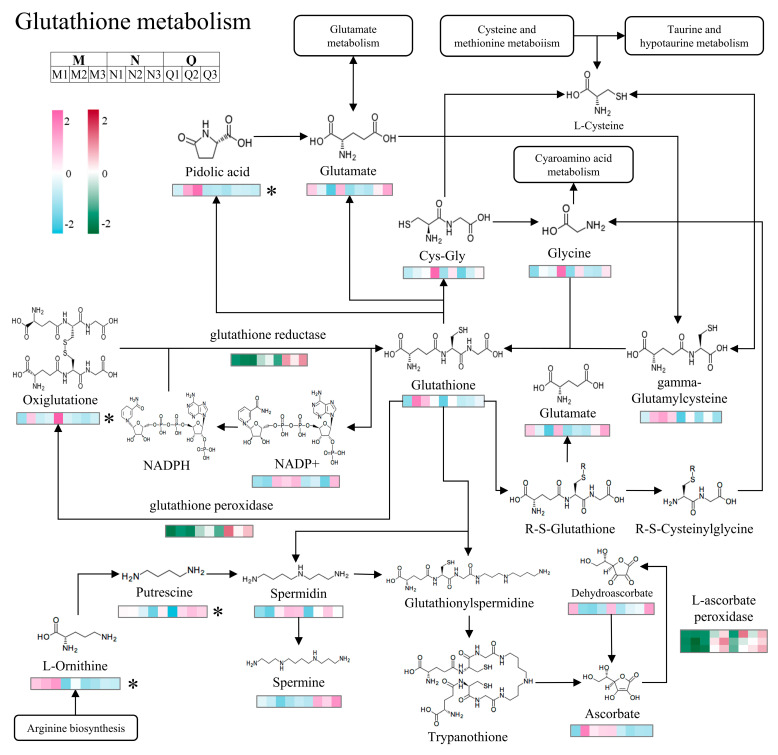
Simplified model of the glutathione metabolic pathway for UV-B versus exogenous ABA treatment. Relevant metabolites and gene content are indicated by heat map bars. For DEGs, redder colors indicate higher relative expression, and greener colors indicate lower. For DMs, the more purple color indicates higher relative expression, and the greener color indicates lower. “*” indicates significant differences.

According to the data, the glutathione metabolism pathway was catalyzed by three important antioxidant enzymes: glutathione reductase, glutathione peroxidase, and L-ascorbate peroxidase (Figure 6). The results show that exogenous ABA further upregulated the DEGs of both enzymes (glutathione reductase and glutathione peroxidase) under UV-B stress, catalyzing the redox reactions involved in oxiglutatione. There was a gradual increase in dehydroascorbate expression and gradual decrease in ascorbate in the glutathione metabolism pathway in the presence of ABA and UV-B. The explanation for this is UV-B and exogenous ABA upregulate the expression of DEGs of L-ascorbate peroxidase, which catalyzes the shift of ascorbate to dehydroascorbate.

### 2.7. Correlation Analysis of DEGs in the GA-Related Pathway with DEGs and DMs in Glutathione Metabolism Pathway

**Figure 7:**
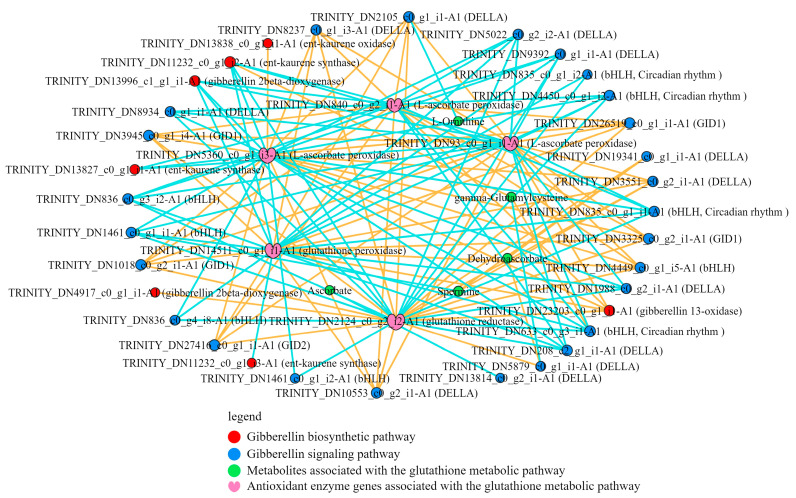
Correlation analysis between UV-B and exogenous ABA treatment of gibberellin biosynthesis and signaling related DEGs with glutathione metabolic pathway related DEGs and DMS. The yellow line represents positive correlation and the blue line represents negative correlation (|R^2^| > 0.8).

Significant changes in GA production and signaling pathways were caused by UV-B and exogenous ABA, while stimulating differential expression of antioxidant enzyme-related genes and significant enrichment in the glutathione metabolism pathway. Therefore, key DEGs and DMs in these pathways were correlatively analyzed to show the connection between them. The results showed that ent-kaurene oxidase, gibberellin 2beta-dioxygenase and gibberellin 13-oxidase, key enzymes in the pathway regulating gibberellin biosynthesis, were significantly correlated with the three key antioxidant enzymes in the pathway regulating the Glutathione metabolism pathway and the metabolic products were significantly correlated (Figure 7, Supplementary Table S4).

Reacting to UV-B, the GA signaling pathway may work in concert with glutathione reductase, glutathione peroxidase, and L-ascorbate peroxidase, indicating a close relationship between the two pathways.

### 2.8. UV-B Causes Acetylation of Key Antioxidant Enzymes in the Glutathione Metabolism Pathway

Transcriptomics and metabolomics studies have shown that glutathione reductase, glutathione peroxidase, and L-ascorbate peroxidase are essential in *R. chrysanthum* in response to UV-B stress. Protein acetylation, as an important post-translational modification, plays an important role in regulating protein function. To further reveal the post-translational modifications of these three proteins of *R. chrysanthum*, this study analyzed them using acetylation proteomics. Non-covalent structures were also visualized using ProteinTools to show the effects of various non-covalent interactions on the structure of the protein in question.

**Figure 8:**
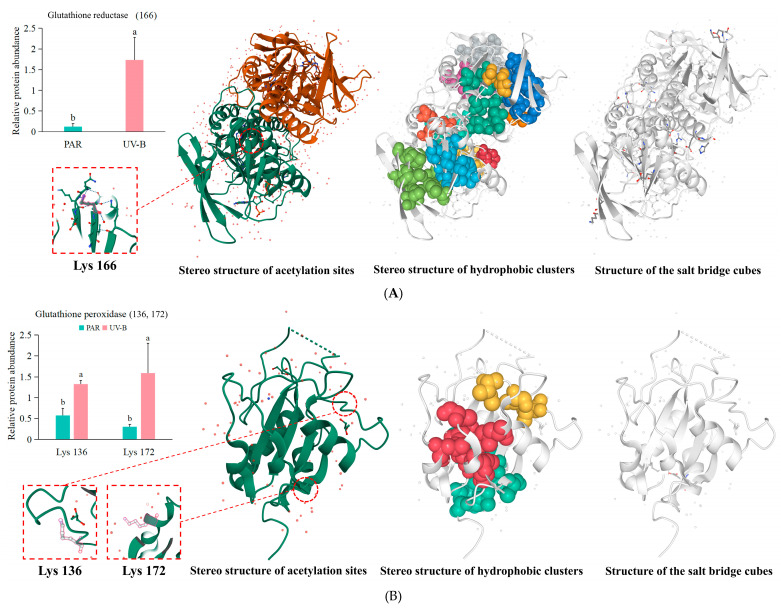

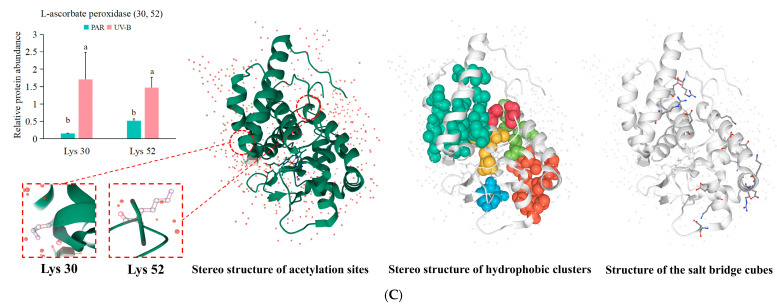
Stereo structure of key antioxidant proteins of *R. chrysanthum*. (**A**) Stereo structure of glutathione reductase of *R. chrysanthum*; (**B**) Stereo structure of glutathione peroxidase of *R. chrysanthum*; (**C**) Stereo structure of L-ascorbate peroxidase of *R. chrysanthum*. The standard deviation is indicated by error bars, whereas the mean of three biological replicates (*n* = 3) is represented by bar heights. Significant differences (*p* < 0.05) are indicated by different lowercase letters.

The results showed that lysine residue 166 of glutathione reductase in the glutathione metabolism pathway underwent significant acetylation modification and was significantly upregulated after UV-B radiation treatment (Figure 8A). There are 14 hydrophobic clusters present in glutathione reductase with hydrophobic cluster areas ranging from 68.71^2^ (Cluster 6) to 2280.57^2^ (Cluster 2). Cluster 6 contains two residues with an area of 34.35^2^ per residue and two interactions between residues. Cluster 2 contains 14 residues with an area of 41.46^2^ per residue and 55 interactions between residues (Supplementary Table S5). Fifteen salt bridge networks were also present in glutathione reductase, and charge separation parameters were calculated. The results showed that the FCR (fraction of charged residues) was 0.23 and K (kappa value) was 0.14 (Supplementary Table S6).

Both glutathione peroxidase and L-ascorbate peroxidase produced two acetylation sites on lysine residues 136 and 172, and lysine residues 30 and 52, respectively (Figure 8B,C). And all these acetylation modifications were significantly upregulated after UV-B. Similar to glutathione reductase, non-covalent interactions like hydrophobic clusters and salt bridges are also present for glutathione peroxidase and L-ascorbate peroxidase (Supplementary Tables S5 and S6).

Taken together, these results indicated that exogenous ABA was able to upregulate the DEGs of glutathione reductase, glutathione peroxidase, and L-ascorbate peroxidase in *R. chrysanthum* in response to UV-B stress. *R. chrysanthum* was also able to further enhance its resistance to UV-B stress through acetylation of these three key antioxidant enzymes.

## 3. Discussion

At different phases of their growth, plants in the natural world may face a range of challenges. After environmental signals are sensed by plants, the environmental response of plants will be regulated through the activation or inhibition of phytohormone pathways and interactions to make adaptive changes [28]. Studies have shown that GA is also involved in plant stress responses to various adversities [29]. The stress resistance mechanism involved in GA mainly includes two forms: one is achieved by inducing the up-regulation of genes such as *GA20ox* and *GA3ox* (Gibberellin 3beta-dioxygenase), which promotes GA synthesis and thus plant growth and development; the other is the upregulation of genes that have the ability to inhibit the activity of GA, such as the expression of genes that inhibit GA activity, such as *GA2ox* (Gibberellin 2beta-dioxygenase), which reduces GA bioactivity, resulting in DELLA accumulation and regulating the downstream pathways [30]. The present study demonstrated the presence of both of these GA stress resistance mechanisms in exogenous ABA-treated *R. chrysanthum* under UV-B stress. UV-B and exogenous ABA resulted in the upregulation of most of the genes involved in GA3 biosynthesis in *R. chrysanthum*, including *GA3ox*, as well as a significant increase in the GA3 content (Figure 1 and Figure 4A). And the light and performance were enhanced by exogenous ABA and GA3 (Figure 2). This implies that GA3 is crucial for *R. chrysanthum*’s retaliation to UV-B and regulated by ABA, whereas exogenous ABA treatment under UV-B stress resulted in a significant decrease in GA53 content (Figure 1). And in the GA53 biosynthesis pathway, UV-B and exogenous ABA caused the significant upregulation of DEG of Gibberellin 13-oxidase. Gibberellin 13-oxidase regulates GA activity and regulates plants by inhibiting GA activity [31]. This is reflected from another perspective that exogenous ABA mainly negatively regulates GA53 and thus opens the GA signaling pathway.

DELLA and GID1 are major components of the GA signaling pathway, in which DELLA plays a key regulatory role and GID1 is a GA receptor [32]. GA is able to cause the degradation of DELLA, which is an inhibitor of plant growth.GA binds to GID1 to form a complex, causing a conformational change in GID1, which then binds to the N-terminus of DELLA, leading to the degradation of DELLA. When GA levels are low, GID1 does not bind to it, and the signaling pathway inhibits the accumulation of the protein DELLA, which stimulates the GA signaling pathway [33]. The present study also found a similar mechanism, with a significant decrease in GA53 due to exogenous ABA, which ultimately led to an increase in the expression levels of most DEGs in DELLA (Figure 4B). Notably, the TFs that were differentially expressed in GA signaling in *R. chrysanthum* were bHLH transcription factors. And some of the differentially expressed bHLH transcription factors are also responsible for regulating the circadian rhythm–plant pathway. Previous studies on *R. chrysanthum* have found that exogenous ABA modulates the Circadian rhythm–plant pathway reacting to UV-B [34]. Combined with the results of this investigation, GA signaling influenced by exogenous ABA is likely to act in conjunction with the Circadian rhythm–plant pathway, and its mechanism deserves further exploration.

Studies have shown that the DELLA protein itself not only resists reactive oxygen molecules induced by abiotic stresses to increase resistance to adversity, but also regulates the antioxidant system of the plant [35,36]. In the present study, we found that UV-B and exogenous ABA enabled the production of a large number of antioxidant DEGs by transcriptomic studies and were significantly enriched in the glutathione metabolic pathway (Supplementary Figure S2). Among them, DEGs, mainly glutathione reductase, glutathione peroxidase, and L-ascorbate peroxidase, were significantly upregulated. Plant glutathione metabolism refers to a series of biochemical processes such as the synthesis, utilization, and regeneration of glutathione (GSH) in plants [37]. Glutathione metabolism not only protects plants from adversity, but also participates in the process of adaptation to environmental changes involving multiple reactive oxygen species scavenging and response to adversity stress. Glutathione reductase can help maintain the reduced state of glutathione. Reduced glutathione (GSH) is one of the most important antioxidants in the cell, scavenging free radicals and reactive oxygen species (ROS) to protect cells from oxidative damage [38]. During photosynthesis in plants, glutathione reductase also helps to protect photosynthetic membranes from ROS damage and maintain photosynthetic efficiency [39,40]. The main role of glutathione peroxidase is to catalyze the generation of oxidized glutathione (GSSG) from reduced glutathione (GSH) to reduce toxic hydrogen peroxide (H_2_O_2_) to non-toxic hydroxyl compounds, thus protecting biofilms from damage caused by reactive oxygen species (ROS) and maintaining normal cellular function [41]. L-ascorbate peroxidase plays a central role in the ascorbate–glutathione (AsA-GSH) cycle in plant cells, catalyzing the decomposition of H_2_O_2_ into water and monodehydroascorbic acid (MDHA) by using ascorbate as an electron donor [42]. This process is essential for maintaining intracellular ascorbic acid and glutathione levels. Thus, exogenous ABA was able to regulate glutathione reductase to maintain the reduced state of GSH, which contributed to the enhancement of cellular antioxidant capacity (Figure 6). Also, exogenous ABA was involved in the regulation of glutathione peroxidase and L-ascorbate peroxidase to scavenge reactive oxygen species caused by UV-B radiation. This is the fundamental reason why exogenous ABA enhances the light and performance of *R. chrysanthum*.

The results of correlation analysis revealed a close correlation between gibberellin biosynthesis and signaling pathways and glutathione metabolism (Figure 7). Combined with the above results, exogenous ABA regulated the gibberellin biosynthesis pathway in *R. chrysanthum* under UV-B stress, which in turn affected the content of gibberellin. Not only that, exogenous ABA also induced the differential expression of gibberellin signaling key component genes, which ultimately turned on the gibberellin signaling pathway. The downstream glutathione metabolic pathway was not only regulated by gibberellin signaling, in which key antioxidant enzyme genes and metabolites were also differentially expressed under the influence of exogenous ABA. Ultimately, these pathways interact with each other and work together to respond positively to UV-B stress.

Protein acetylation modification, as a post-translational modification, involves the addition of an acetyl group to a specific amino acid residue of a protein, usually the ε-amino side chain of lysine (Lysine) [43]. Protein stability may be impacted by acetylation. Moreover, acetylation frequently results in gene activation, which modifies the activity of enzymes. Hydrophobic clusters figure prominently in the stabilization of protein structures by promoting the aggregation of hydrophobic residues in the interior of the protein molecule, while hydrophilic residues are located on the surface of the molecule [44]. Salt bridge in proteins is an important noncovalent interaction that involves electrostatic interactions between oppositely charged amino acid residues in a protein molecule [45]. The formation of salt bridges helps to stabilize the three-dimensional structure of proteins and is critical for protein functions, including enzyme catalysis, protein-protein interactions, and molecular recognition. In the present study, it was found that UV-B radiation could indeed further lead to acetylation modification of glutathione reductase, glutathione peroxidase, and L-ascorbate peroxidase (Figure 8). This corroborates the transcriptomics results that the genes for the three core antioxidant enzymes are activated. The question of how the acetylation modification of these antioxidant enzymes affects their own activities and the role played by non-covalent bonds in them deserves further investigation.

## 4. Materials and Methods

### 4.1. Treatment and Culture of Experimental Plants

Reference was made to previous experiments regarding the culture conditions and experimental treatments of the material used in this experiment [46,47]. In this experiment, *R. chrysanthum* was the experimental material. It was cultivated in an incubator with a photon flux density of 50 μmol/(m^2^s) and a temperature of 25/18 °C with a 10/14 h day/night cycle. High transmission filters with various transmittance properties were utilized to obtain the diverse radiation conditions needed. The 400 nm wavelength filters (Edmund, Filter Long 2IN SQ, Barrington, NJ, USA) were placed on the culture flasks for PAR (photosynthetically active radiation) irradiation only. UV-B radiation treatment was carried out by placing 295 nm wavelength filters(Edmund, Filter Long 2IN SQ, Barrington, NJ, USA) on each of the culture flasks for UV-B stress. Warm white fluorescent lights (Philips, T5 14W, Amsterdam, Netherlands) were used to produce visible light. UV-B radiation was provided by UV-B fluorescent tubes (Philips, Ultraviolet-B TL 20W/01 RS, Amsterdam, The Netherlands). The effective irradiance of the samples was 2.3 W/m^2^ for UV-B and 50 μmol/(m^2^s) for PAR.

For the experiment, eight-month-old *R. chrysanthum* in the same growth stage was chosen. A total of four groups of treatments were created, which were recorded as M, N, Q, and GA, and three biological replications were set up for each group of treatments (*n* = 3). *R. chrysanthum*s were cultivated in groups M and N utilizing 1/4 MS medium. The Q and GA groups were cultured in 1/4 MS medium supplemented with ABA (100 µM) and GA3 (100 µM), respectively, and were placed in an artificial climate chamber for 7 d after transplanting. Group M was subjected to PAR treatment for 8 h/d for 2 d. Groups N, Q and GA were subjected to PAR+UV-B treatments with the same treatment duration as in Group M (Table 1). The experimental treatment of each group was completed, after which the determination of the indicators was carried out.

### 4.2. Determination of Widely Targeted Metabolomics and Plant Hormones

This part was completed by Metware Biotech Inc., Wuhan, China. The manipulation technique, equipment, and reagents were all the same as in the earlier studies [48]. The following are the primary steps:

Multiquant and Analyst 1.6.3 were used to perform both qualitative and quantitative analyses of the plant metabolites. First, the metabolites in the samples were identified by a self-constructed MWDB database, combining with primary and secondary mass spectrometry data. The metabolites were then measured with triple quadrupole mass spectrometry operating in the MRM mode. By applying integral corrections to the chromatographic peaks and comparing the mass spectral signals in various samples, quantitative information about metabolites was acquired. Lastly, by establishing the criteria of fold change (FC) over 1.5 or below 0.67 and VIP values more than 1, statistically differential metabolites (DMs) were obtained. These DMs were further annotated to the KEGG database for subsequent analysis.

Liquid chromatography–tandem mass spectrometry (LC-MS/MS) was used to determine phytohormones, strictly adhering to established experimental protocols [49]. Jingjie PTM Biolab completed the phytohormone assay for this experiment.

### 4.3. Determination of Transcriptomics

Transcriptomics experiments were performed by Huada Gene Science and Technology Research Co., Shenzhen, China. The apparatus and reagents, as well as the method of operation, are identical to those of the previous experiment [50]. The following are the primary steps:

The whole RNA was processed and purified using the CTAB technique. After that, single-stranded circular DNA libraries were created, and at last, high-throughput sequencing was applied. SOAPnuke (v1.4.0) was used for filtering in order to obtain precise sequencing data. The clean reads were de novo assembled by Trinity, and the assembled transcripts were subsequently clustered and de-redundant using CD-HIT, thereby obtaining unigenes. The assembled data were annotated in KEGG, GO, NR, NT, Pfam, KOG and SwissProt.

Bowtie2 was used to compare clean reads to reference gene sequences. Each gene’s and transcript’s expression levels were then determined. The FPKM method was utilized to determine the differentially expressed genes (DEGs) present in *R. chrysanthum* leaves under both normal and UV-B stress. Each transcript’s gene expression was then calculated. The thresholds of Qvalue (adjusted *p* value) < 0.05 and FC (fold change) > 1 were applied to assess the significance of the variations in gene expression.

### 4.4. Determination of Acetylated Proteomics

Quantitative proteomic assays for acetylation modifications in this study were performed by Jingjie PTM Biolab, Zhejiang, China. The apparatus and reagents, as well as the method of manipulation, followed the existing experiments exactly [12]. The following are the primary steps:

First, plant leaf proteins were extracted from plants that had undergone specific experimental treatments. The relevant protein content was determined using a two-dimensional quantitative analysis kit. Next, enzymatic digestion was performed using trypsin, and the resulting peptides were desalted by a Strata X SPE column. Afterwards, the peptides were further desalted using C18 ZipTips (Millipore, Burlington, ON, Canada) according to the manufacturer’s guidelines. After completing the affinity enrichment of peptides, LCMS/MS analysis was performed. Peptides were subjected to capillary source treatment before analysis on a timsTOF Pro (Bruker Dalton, Billerica, MA, USA) mass spectrometer. Next, the analyzed mass spectrometry data were subjected to in-depth analysis by the MaxQuant search engine (1.6.6.0) and protein annotation in KEGG database. During the experiments, protein N-terminal acetylation, methionine (Met) oxidation, and lysine (Lys) acetylation were defined as variable modifications, whereas aminocarbonamide on cysteine (Cys) was used as a fixed modification. Ultimately, a false discovery rate (FDR) threshold of less than 1% was set and a *p*-value of less than 0.05 and at least 1.5-fold fold change (FC) were used as screening criteria for differential proteins.

A total of 807 differentially expressed proteins (450 increased in abundance and 357 decreased in abundance) were obtained after UV-B radiation. And 685 acetylated differentially expressed proteins (95 increased in abundance and 590 decreased in abundance) and 945 acetylation sites (104 upregulated and 841 downregulated) were out after UV-B radiation.

Modeling of acetylated protein homology followed previous descriptions [12]. NCBI BLAST was used to look for homologous sequences. Next, the comparative protein modeling platform SWISS-MODEL (https://swissmodel.expasy.org/ (accessed on 6 May 2024)) was utilized to generate a three-dimensional structural model of the acetylated protein.

### 4.5. Determination of Chlorophyll Fluorescence Parameters

The IMAGING-PAM chlorophyll fluorescence imaging equipment (Heinz Walz, Effeltrich, Germany) was used to calculate the parameters of chlorophyll fluorescence. At the moment of determination, three observation spots were chosen on the leaves of the chosen test materials, and the experiment was run for 30 min following dark adaptation. Table 2’s pertinent parameters were ascertained.

### 4.6. Data Analysis

Each of the aforementioned experiments was conducted three times, employing a completely randomized experimental design. IBM SPSS was used to conduct the significance analysis (IBM SPSS Statistics 26). One-way analysis of variance (ANOVA) was used as the analysis method. The Pearson correlation test was used to measure correlation, and a 5% cutoff was used. The HCA (hierarchical cluster analysis) visualization was created with the ComplexHeatmap R package (2.9.4). Plotting was carried out using the R package MetaboAnalystR (1.0.1) for orthogonal partial least squares–discriminant analysis (OPLS-DA). It was log2-converted and then centered prior to plotting. The KEGG compound database (http://www.kegg.jp/kegg/compound/ (accessed on 6 May 2024)) was used to annotate metabolites.

## 5. Conclusions

In this study, *R. chrysanthum* treated with UV-B and exogenous ABA was analyzed jointly in a multiomics approach. The results showed that exogenous ABA led to changes in the content of *R. chrysanthum* gibberellins and contributed to the upregulation of antioxidant genes. Exogenous ABA enhanced the resistance of *R. chrysanthum* to UV-B stress by regulating gibberellin signaling and antioxidant enzyme gene expression. In addition, acetylation of key antioxidant enzymes under UV-B exposure was also important for the positive response of *R. chrysanthum* to UV-B stress. The results of the findings highlight the role of ABA in plant stress tolerance and provide insights for the development of stress-tolerant plant varieties.

## Figures and Tables

**Table 1:** Treatment of experimental materials.

Groups	Culture Medium	Radiation Mode	Radiation Duration
M	1/4 MS	PAR 50 μmol/(m^2^s)	Two days, 8 h per day
N	1/4 MS	PAR 50 μmol/(m^2^s) + UV-B (2.3 W/m^2^)	
Q	1/4 MS + ABA (100 µM)	PAR 50 μmol/(m^2^s) + UV-B (2.3 W/m^2^)	
GA	1/4 MS + GA3 (100 µM)	PAR 50 μmol/(m^2^s) + UV-B (2.3 W/m^2^)	

**Table 2:** Chlorophyll fluorescence parameters and calculation formulae.

Chlorophyll Fluorescence Parameters	Formulas
Y(II) (Photochemical yield of PSII)	(Fm’ − F)/Fm’
Fv/Fm (Maximal photochemical efficiency of PSII)	(Fm − Fo)/Fm
ETR (actual electron transport rate)	PPFD·ΦPSII·0.85·0.5
NPQ (non-photochemical quenching)	(Fm − Fm′)/Fm′
qL (photochemical quenching)	1 − (Fm′ − Fo′)/(Fm − Fo)

## Data Availability

The data used in this study are available from the corresponding author on submission of a reasonable request.

## Supplementary Materials

The following supporting information can be downloaded at: https://www.mdpi.com/article/10.3390/ijms252413651/s1.
