# Supplementary material for: Regulatory Mechanism of Exogenous ABA on Gibberellin Signaling and Antioxidant Responses in Rhododendron chrysanthum Pall. Under UV-B Stress
Source: Int J Mol Sci. 2024 Dec 20;25(24):13651. doi: 10.3390/ijms252413651 (PMC11728028; doi:10.3390/ijms252413651)
Supplement: Supplementary file 1 [file ijms-25-13651-s001.zip › Supplementary Figures.pdf]

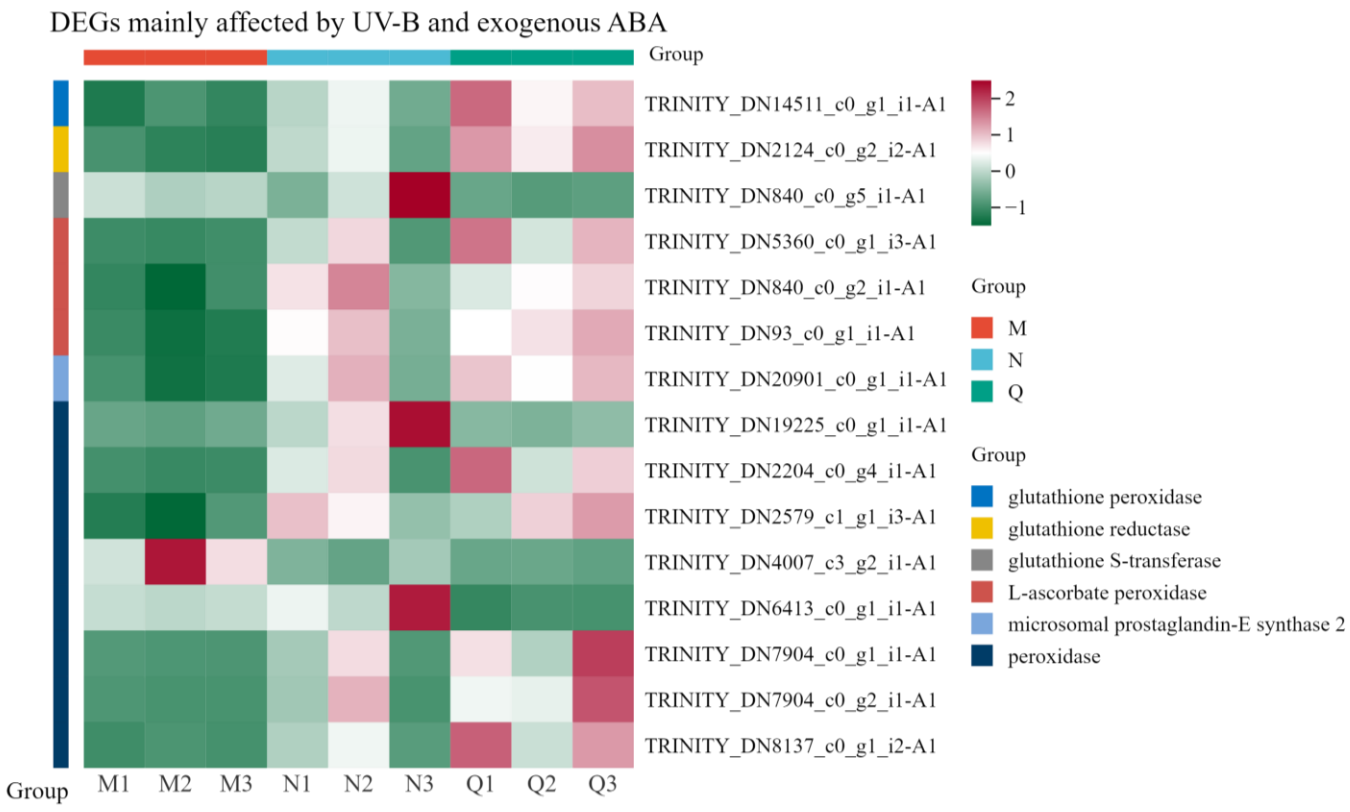

Figure S1. Clustering heatmap of 15 overlapping DEGs under UV-B and exogenous ABA treatments. The expression of the relevant DEGs in the figure is indicated by color. For DEGs, redder colors indicate higher relative expression, and greener colors indicate lower expression.

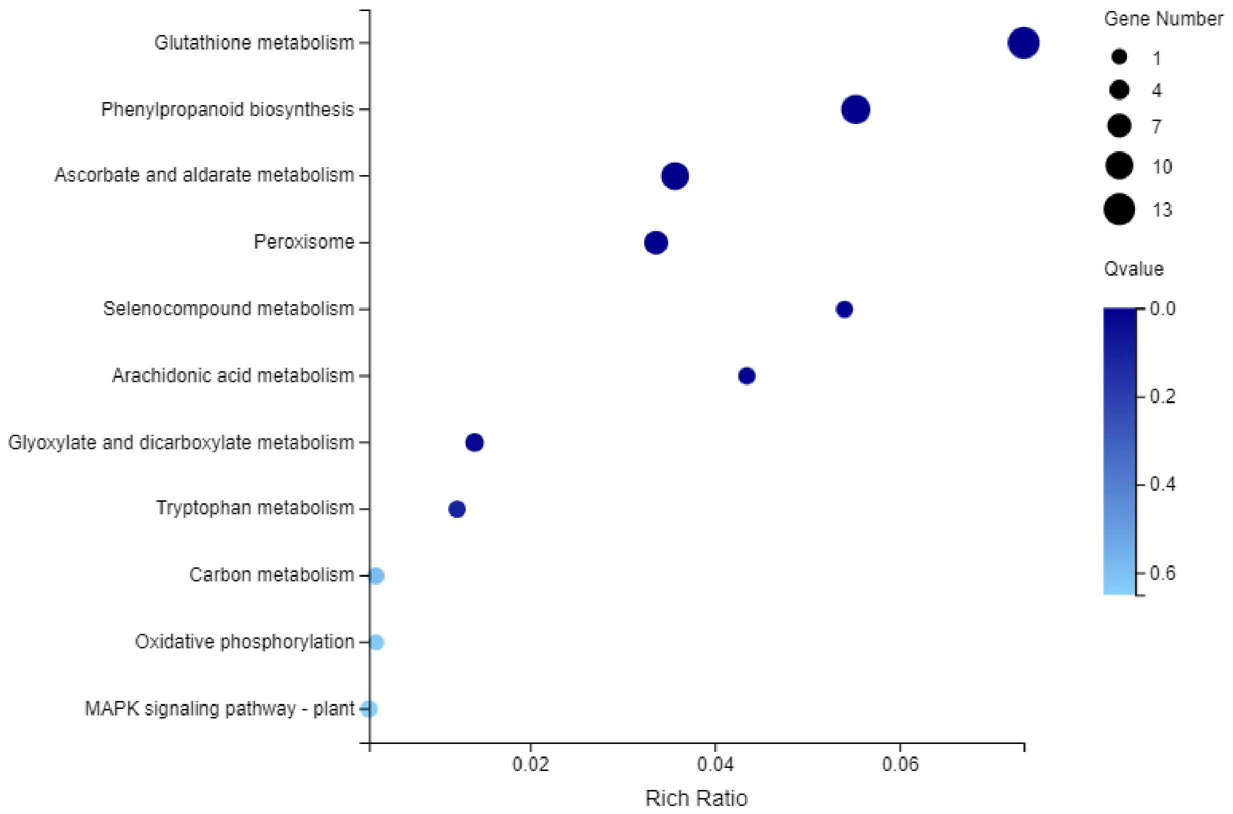

Figure S2. KEGG enrichment analysis of 15 recombinant DEGs under UV-B and exogenous ABA treatments. Bubble size responds to the number of DEGs. Significance was reflected by bubble color, the darker the color the more significant.

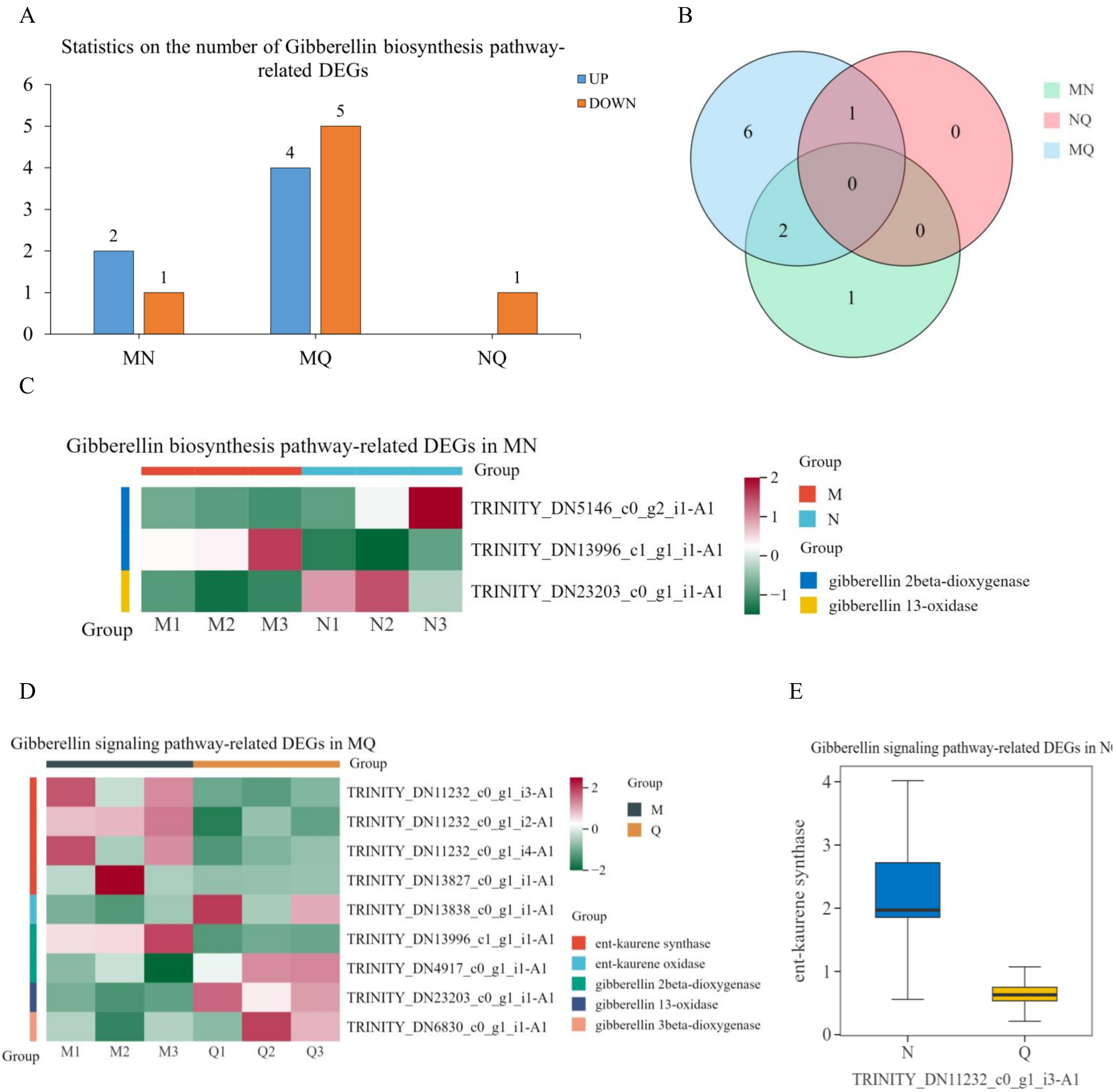

Figure S3. Changes of gibberellin biosynthesis pathway-related genes under UV-B and exogenous ABA treatments. (A) Statistics of the number of gibberellin biosynthesis pathway-related DEGs in each comparative group; (B) Wayne's plots of gibberellin-resistant biosynthesis pathway-related DEGs in each comparative group; (C-D) Clustering heatmaps of gibberellin biosynthesis pathway-related DEGs in each comparative group. The expression of relevant DEGs in the graphs is indicated by color. Redder colors indicate higher relative expression, and greener colors indicate lower expression. (E) Box plot of DEGs in the NQ group. The bolded black line in the middle of the box plot indicates the median, and the topmost and least-bottomed margins indicate the maximum and minimum values, respectively.

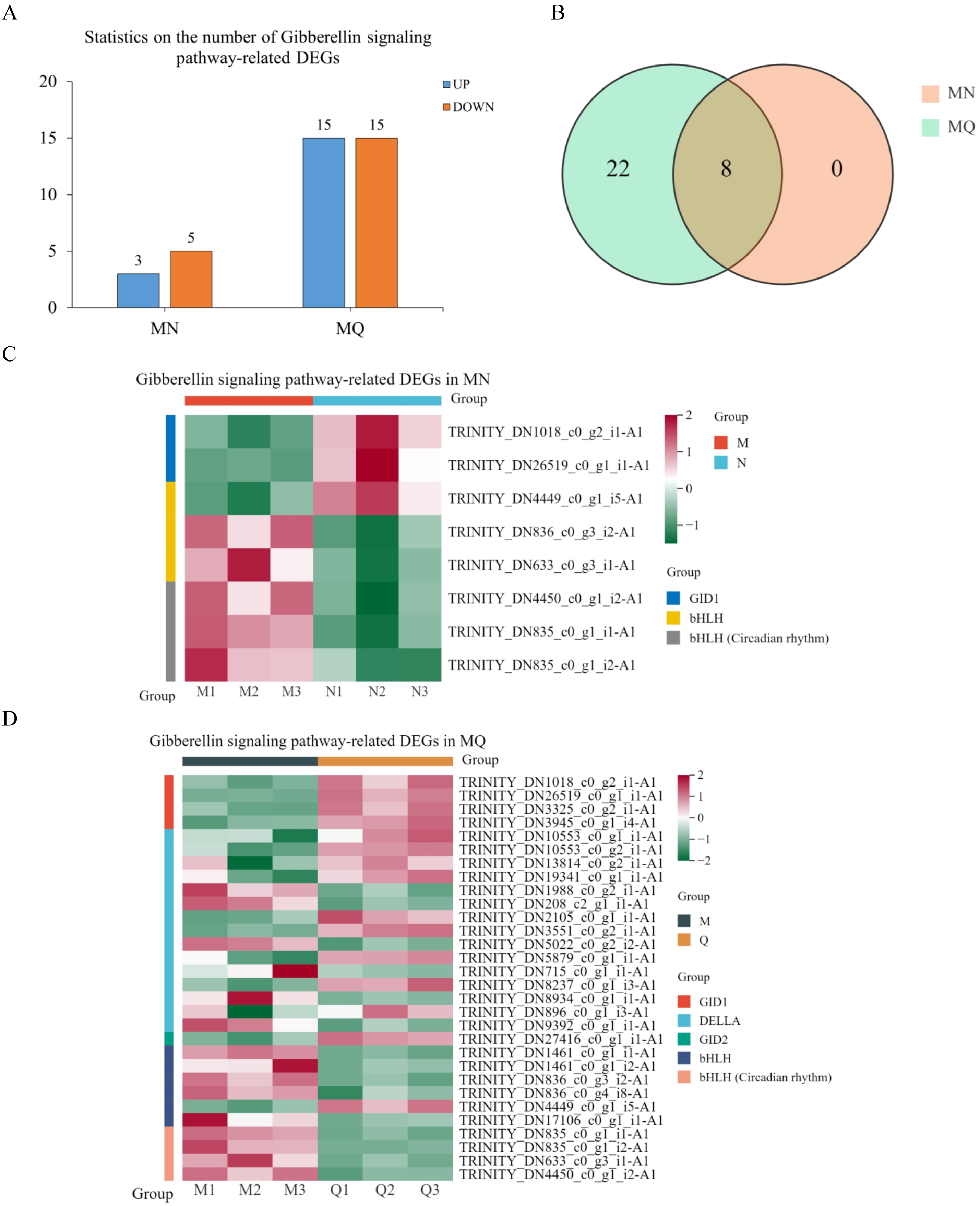

Figure S4. Changes of genes related to the gibberellin signaling pathway under UV-B and exogenous ABA treatments. (A) Statistics on the number of DEGs related to the gibberellin signaling pathway in each comparison group; (B) Wayne plots of the DEGs related to the gibberellin signaling pathway in each comparison group; (C-D) Clustering heatmaps of the DEGs related to the gibberellin signaling pathway in each comparison group. The expression of relevant DEGs in the graphs is indicated by color. Redder colors indicate higher relative expression, and greener colors indicate lower expression.

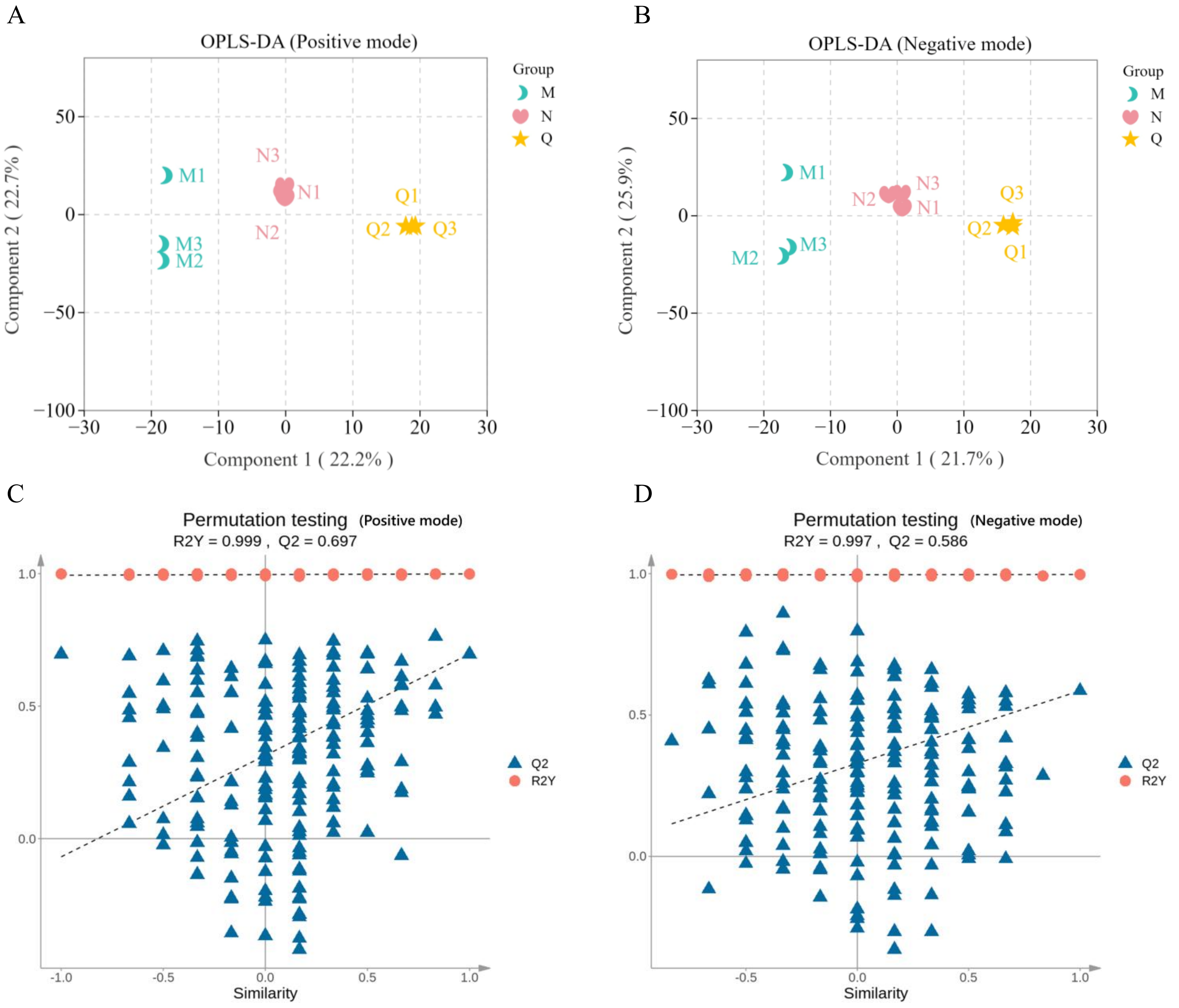

Figure S5.OPLS-DA analysis. (A, B) OPLSDA analysis of metabolites detected in different scanning methods; (C, D) OPLSDA validation model, which is considered valid when Q2>0.5.
